# Drug-Target Interaction Prediction via Dual Laplacian Graph Regularized Logistic Matrix Factorization

**DOI:** 10.1155/2021/5599263

**Published:** 2021-03-26

**Authors:** Aizhen Wang, Minhui Wang

**Affiliations:** ^1^Department of Pharmacy, The Affiliated Huai'an Hospital of Xuzhou Medical University and The Second People's Hospital of Huai'an, Huai'an 223002, China; ^2^Department of Pharmacy, Lianshui People's Hospital Affiliated to Kangda College, Nanjing Medical University, Huai'an 223300, China

## Abstract

Drug-target interactions provide useful information for biomedical drug discovery as well as drug development. However, it is costly and time consuming to find drug-target interactions by experimental methods. As a result, developing computational approaches for this task is necessary and has practical significance. In this study, we establish a novel dual Laplacian graph regularized logistic matrix factorization model for drug-target interaction prediction, referred to as DLGrLMF briefly. Specifically, DLGrLMF regards the task of drug-target interaction prediction as a weighted logistic matrix factorization problem, in which the experimentally validated interactions are allocated with larger weights. Meanwhile, by considering that drugs with similar chemical structure should have interactions with similar targets and targets with similar genomic sequence similarity should in turn have interactions with similar drugs, the drug pairwise chemical structure similarities as well as the target pairwise genomic sequence similarities are fully exploited to serve the matrix factorization problem by using a dual Laplacian graph regularization term. In addition, we design a gradient descent algorithm to solve the resultant optimization problem. Finally, the efficacy of DLGrLMF is validated on various benchmark datasets and the experimental results demonstrate that DLGrLMF performs better than other state-of-the-art methods. Case studies are also conducted to validate that DLGrLMF can successfully predict most of the experimental validated drug-target interactions.

## 1. Introduction

It is well known that drug discovery is a difficult and expensive process, and identifying potential drug-target interactions (DTIs) plays an important role in yielding successful candidate compounds for drug development. Predicting interactions between different drugs and targets can provide critical information by discovering off-target effects. Accurate prediction of DTIs can also substantially accelerate lead generation. Drug-target interaction prediction can be also regarded as a useful step in biomedical research and precision medicine [1–9]. However, it is still time consuming for traditional experimental approaches to identify potential DTIs, and the success rates are also very low. In addition, only a very limited number of DTIs have been experimentally validated. Therefore, it is necessary to develop computational methods for DTIs, which can significantly reduce both the time and labor costs, as well as improve the efficiency of drug discovery. Furthermore, there are various datasets which contain experimentally validated interactions of drugs and targets, such as KEGG [10], DrugBank [11], and GenBank [12], which also benefit the prediction of potential DTIs by using computational techniques.

In recent years, a large number of computational methods for DTI prediction have been proposed, and these methods are often based on some machine learning and data mining models, e.g., logistic regression [13, 14], support vector machine (SVM) [15–17], Bayesian classifiers [18], matrix completion [9], matrix factorization [19, 20], kernel learning [21, 22], and network inference [23–25]. For classification-based methods, they treat drug-target interaction pairs and noninteraction pairs as positive instances or negative instances and convert the DTI prediction problem into a label classification task [14, 17]. In [15], a genetic algorithm is used to screen related compounds; the drug-target pairs with strong binding capacity were found with SVM and particle swarm optimization. Garcasosa et al. [13, 18] used logistic regression and naive Bayesian classifiers for the classification of compounds. In [26], the experimentally validated targets are employed to train a SVM model and find potential proteins with similar structure. Network-based methods [23, 27, 28] utilize the theory of network and graph [29] and incorporate the drug and target similarities with experimentally validated interactions to infer the potential unknown drug-target interactions. Due to the strong learning capability of data representation, matrix factorization has also been used for drug-target interaction prediction, such as Bayesian matrix factorization [30], collaborative matrix factorization [31], and robust graph regularized matrix factorization [20]. The principle of these matrix factorization-based methods lies in that a high-dimensional drug-target interaction matrix can be decomposed into a multiplication of low-dimensional matrices, and the intrinsic property of original data can be well captured by these low-dimensional matrices. Due to the powerful feature representation and linear relation learning capability, deep neural network-based methods are also proposed to learn the relationship between drug and target [32, 33].

Although achieving great success by previous computational model learning-based methods, there are still some limitations and much room for improvement. Firstly, in previous methods, the experimentally validated and unknown interactions are treated equally during the learning process, which could deduce some noisy information. Secondly, in some learning models, drug features and target features are difficult to select. In order to solve the above issues, we propose a novel drug-target interaction prediction model based on logistic matrix factorization with dual Laplacian graph regularization term by using experimentally validated interactions, referred to as DLGrLMF briefly. In our DLGrLMF model, the chemical structure similarities between drug pairs, the genomic sequence similarities between target pairs, and the experimentally validated interactions are integrated together. The similarities between drug neighbors and target neighbors are exploited to represent the latent factor vector of the factorized matrices, and the potential interactions are determined by a probability score through the logic function.

The efficacy of the proposed DLGrLMF was evaluated on five benchmark datasets, and we compared DLGrLMF with several other state-of-the-art drug-target interaction prediction approaches in terms of 10-fold cross-validation, and the results demonstrate that the proposed DLGrLMF clearly outperforms other methods. In addition, in order to validate the ability to predict potential drug-target interactions, case studies are also performed and the results also demonstrate that DLGrLMF can accurately predict most of the experimental validated drug-target interactions.

## 2. Materials and Methods

### 2.1. Datasets Used in Experiments

In this work, four small-scale benchmark datasets and a large-scale dataset are used in the experiments to evaluate the DTI prediction performance of the proposed DLGrLMF model. The four small-scale datasets include nuclear receptors (NRs), G protein-coupled receptors (GPCRs), ion channels (ICs), and enzymes (Es) [34], there are four different types of target protein, and they are publicly available at http://web.kuicr.kyoto-u.ac.jp/supp/yoshi/drugtarget/. As to the large-scale dataset DrugBank (DB) [35], it is a unique bioinformatics and cheminformatics resource which combines detailed drug data with comprehensive drug-target information. We use the data released on Jul. 03, 2018 (version 5.1.1), in our experiments. The drug and target data were extracted from the DrugBank database website at http://www.drugbank.ca/. We only use the approved drug-target interactions for experiments. To this end, there are totally 1936 drugs, 1609 targets, and 7019 approved drug-target interactions, respectively. We download the approved drug structures and approved target sequences from https://www.drugbank.ca/releases/latest#structures and https://www.drugbank.ca/releases/latest#target-sequences, respectively.

In Table 1, we present the detailed statistics of the five datasets. Three types of information for each dataset are summarized, including the similarities between drug pairs, the similarities between target pairs, and the experimental validated DTIs. Specifically, the validated DTIs are obtained from public datasets including KEGG BRITE [36], BRENDA [37], DrugBank [38], and SuperTarget [39].

### 2.2. Problem Formulation of DTI Prediction

In order to make the subsequent expression clearer, we first give a brief problem formulation of DTI prediction. Throughout this paper, we use two sets *𝒯* = {*T*_*i*_}_*i*=1_^*t*^ and *𝒟* = {*D*_*i*_}_*i*=1_^*d*^ to represent *t* targets and *d* drugs, respectively. The experimentally validated DTIs are denoted as a binary matrix **A** ∈ {0, 1}^*d*×*t*^. If a drug *D*_*i*_ has been experimentally validated to interact with a target *T*_*j*_, then *A*_*ij*_ = 1; otherwise, *A*_*ij*_ = 0. The elements with a value of “1” in **A** represent the “known interactions” and can be regarded as positive observations, while the zero elements in **A** are set as “unknown interactions” and can be regarded as negative observations. In addition, the drug similarities are denoted as **DS** ∈ ℝ^*d*×*d*^, and the target similarities are represented as **TS** ∈ ℝ^*t*×*t*^. DTI prediction is aimed at discovering the potential interactions from the negative observations by using certain prior information of drugs and targets. The candidate drug-target interactions will be chosen as predicted interactions according to their predicted probabilities in descending order.

For each dataset, three matrices including **A**, **DS**, and **TS** are provided, which represent the drug-target interactions, drug similarities, and target similarities, respectively. Each entry of **DS** represents the similarity between a drug pair, which is measured by using SIMCOMP [40] that describes the chemical structure similarity between drugs. In SIMCOMP, the similarity between two compounds *c*1 and *c*2 can be computed as *S*(*c*1, *c*2) = ∣*c*1∩*c*2 | /∣*c*1 ∪ *c*2∣. As to **TS**, genomic sequence similarity is used to denoting the similarity score between two proteins, which are obtained from the KEGG GENES dataset [36]. The sequence similarities between two proteins *p*1 and *p*2 are computed via a normalized version of the Smith–Waterman scores [41], which is defined as $S\left(p1,p2\right)=SW\left(p1,p2\right)/\sqrt{SW\left(p1,p1\right)SW\left(p2,p2\right)}$, where *SW*(·, ·) represents the original unnormalized Smith-Waterman score.

### 2.3. Proposed DLGrLMF Model

Matrix factorization has been developed for recommendation systems in the very beginning, which decomposes the observation data matrix **X** ∈ *ℛ*^*m*×*n*^ into two low-dimensional matrices **U** ∈ *ℛ*^*m*×*k*^ and **V** ∈ *ℛ*^*n*×*k*^, where *k* is the so-called number of hidden factors. Then, **U** and **V** can be regarded as the latent representation of drugs and targets in the hidden space. In recent years, it has also been used for predicting the incRNA-miRNA relationship [42] and drug-target interaction [20]. In this work, we propose a drug-target interaction prediction model via logistic matrix factorization with dual Laplacian graph regularization. Here, the occurrence probability *p*_*ij*_ of a certain drug-target interaction is calculated based on the inner product of the latent factor vectors from drug and target. Specifically, *p*_*ij*_ can be formulated as follows:

(1) $$\begin{array}{c} p_{ij}=\frac{e^{\left(u_{i}{v_{j}}^{T}\right)}}{\left(1+e^{\left(u_{i}{v_{j}}^{T}\right)}\right)}, \end{array}$$

where *u*_*i*_ is a latent row vector to represent drug *d*_*i*_, and *v*_*j*_ is a latent row vector to represent target *t*_*j*_. Then, *U* and *V* can be used to represent the potential characteristics of all drugs and targets, respectively. In this work, **U** and **V** are initialized by zero-mean spherical Gaussian priors as follows:

(2) $$\begin{array}{c} p\left(U\;|\;{\sigma_{u}}^{2}\right)=\overset{d}{\underset{i=1}{\prod }}N\left(u_{i}\;|\;0,{\sigma_{u}}^{2}I\right),\\ p\left(V\;|\;{\sigma_{v}}^{2}\right)=\overset{t}{\underset{j=1}{\prod }}N\left(v_{j}\;|\;0,{\sigma_{v}}^{2}I\right), \end{array}$$

where *N*(*x*_*i*_ | *z*, **Z**) represents a Gaussian probability density function with a mean of *z*, a variance of **Z**, and an independent variable *x*_*i*_.In drug-target interaction prediction studies, known DTIs are experimentally verified and they should be more reliable than unknown relationships. Therefore, we should allocate higher weights to those known DTIs [43, 44]. Specifically, the known DTI pairs and *n*(*n* > 1) negative samples are used for training, where the constant *n* determines the significance of the interaction pairs. Then, the posterior distribution in logarithm can be written as follows:

(3) $$\begin{array}{c} \log p\left(U,V\;|\;A,{\sigma_{u}}^{2},{\sigma_{v}}^{2}\right)=\overset{d}{\underset{i=1}{\sum }}\overset{t}{\underset{j=1}{\sum }}na_{ij}u_{i}{v_{j}}^{T}-\left(1+na_{ij}-a_{ij}\right)\log\left[1+e^{\left(u_{i}{v_{j}}^{T}\right)}\right]-12{\sigma_{u}}^{2}\overset{d}{\underset{i=1}{\sum }}{\left\|u_{i}\right\|}_{2}^{2}-12{\sigma_{v}}^{2}\overset{t}{\underset{j=1}{\sum }}{\left\|v_{j}\right\|}_{2}^{2}+C, \end{array}$$

where *C* is a constant.

Although minimizing Equation (3) can exploit latent vectors globally to predict potential DTIs, the local similarity information implied among drugs and targets is not taken into consideration. Therefore, we use the drug similarities and the target similarities to boost the prediction performance. Instead of using all of the similarities in **DS** and **TS**, we extract the local neighbor information of drugs and targets. As to different drugs, the local neighbor similarity matrix $\bar{DS}$ can be obtained from **DS** by

(4) $$\begin{array}{c} \bar{DS}\left(ij\right)=\left\{\begin{array}{cc} DS\left(ij\right), & \text{if}\;d_{i}\in N\left(d_{j}\right),\\ 0, & \text{otherwise}, \end{array}\right. \end{array}$$

where *𝒩*(*d*_*j*_) denoted the neighbors of drug *d*_*j*_.In a similar way, the local neighbor similarity matrix $\bar{TS}$ can be obtained from **TS** by

(5) $$\begin{array}{c} \bar{TS}\left(ij\right)=\left\{\begin{array}{cc} TS\left(ij\right), & \text{if}\;t_{i}\in N\left(t_{j}\right),\\ 0, & \text{otherwise}, \end{array}\right. \end{array}$$

where *𝒩*(*t*_*j*_) denoted the neighbors of target *t*_*j*_.Since the iterations between drugs and targets are complex, different to previous graph regularized methods that only use the first-order connections to reflect the local pairwise proximity between vertices in a graph [45–48], we use the second-order connection to constrain that similar drugs should be connected with similar targets. Therefore, we have the following similarity affinity matrices calculating form:

(6) $$\begin{array}{c} \bar{DS}\left(ij\right)=\bar{DS}\left(i\right)\bar{DS}{\left(j\right)}^{T},\\ \bar{TS}\left(ij\right)=\bar{TS}\left(i\right)\bar{TS}{\left(j\right)}^{T}, \end{array}$$

where $\bar{DS}\left(i\right)$ and $\bar{DS}\left(j\right)$ represent the *i*th and *j*th column of original $\bar{DS}$, respectively, and $\bar{TS}\left(i\right)$ and $\bar{TS}\left(j\right)$ represent the *i*th and *j*th column of original $\bar{TS}$, respectively.

The main idea of our proposed DLGrLMF model is under the assumption that if the chemical structure of two drugs from a drug pair is similar to each other, their latent representation should also be closed to each other. Similarly, the latent representation of two targets should also be similar to each other if their genomic sequence similarities are closed to each other. For drugs, we can minimize the following problem:

(7) $$\begin{array}{c} \underset{U}{\min}\overset{d}{\underset{i=1}{\sum }}\overset{d}{\underset{j=1}{\sum }}\bar{DS}\left(ij\right){\left\|u_{i}-u_{j}\right\|}_{2}^{2}. \end{array}$$

As to different targets, we have the following similar minimization problem:

(8) $$\begin{array}{c} \underset{V}{\min}\overset{t}{\underset{i=1}{\sum }}\overset{t}{\underset{j=1}{\sum }}\bar{TS}\left(ij\right){\left\|v_{i}-v_{j}\right\|}_{2}^{2}. \end{array}$$

By some simple algebra, Equations (7) and (8) can be transformed into the following form:

(9) $$\begin{array}{c} \underset{U}{\min}Tr\left(U^{T}L_{D}U\right), \end{array}$$

(10) $$\begin{array}{c} \underset{V}{\min}Tr\left(V^{T}L_{T}V\right), \end{array}$$

where **L**_**D**_ ∈ ℝ^*d*×*d*^ is the corresponding Laplacian matrix of drugs with $L_{D}=D_{D}-\bar{DS}$, and **D**_**D**_ is the diagonal matrix with $D_{D}\left(i,i\right)=\sum_{j}\bar{DS}\left(i,j\right)$. **L**_**T**_ ∈ ℝ^*t*×*t*^ is the corresponding target Laplacian matrix of targets with $L_{T}=D_{T}-\bar{TS}$, and **D**_**T**_ is the diagonal matrix with $D_{T}\left(p,p\right)=\sum_{q}\bar{TS}\left(p,q\right)$.By combining Equations (9) and (10) and the maximization of Equation (3) together, we have our final DLGrLMF model as follows:

(11) $$\begin{array}{c} \underset{U,V}{\min}\left(1+na_{ij}-a_{ij}\right)\log\left[1+e^{\left(u_{i}{v_{j}}^{T}\right)}\right]-\overset{d}{\underset{i=1}{\sum }}\overset{t}{\underset{j=1}{\sum }}na_{ij}u_{i}{v_{j}}^{T}+12{\sigma_{u}}^{2}\overset{d}{\underset{i=1}{\sum }}{\left\|u_{i}\right\|}_{2}^{2}+12{\sigma_{v}}^{2}\overset{t}{\underset{j=1}{\sum }}{\left\|v_{j}\right\|}_{2}^{2}+Tr\left(U^{T}L_{D}U\right)+Tr\left(V^{T}L_{T}V\right), \end{array}$$

which is equal to the following problem:

(12) $$\begin{array}{c} \underset{U,V}{\min}\left(1+na_{ij}-a_{ij}\right)\ln\left[1+e^{\left(u_{i}{v_{j}}^{T}\right)}\right]-\overset{d}{\underset{i=1}{\sum }}\overset{t}{\underset{j=1}{\sum }}na_{ij}u_{i}{v_{j}}^{T}+\alpha Tr\left(U^{T}\left(L_{D}+I_{m}\right)U\right)+\beta Tr\left(V^{T}\left(L_{T}+I_{n}\right)V\right), \end{array}$$

where **I**_*m*_ and **I**_*n*_ represent two identity matrices with size *m* × *m* and *n* × *n*, respectively. *α* and *β* are two nonnegative constants to balance the regularization terms. In Equation (12), the first and second terms constitute the logistic matrix factorization model to formulate the drug-target interaction probability. The third term is the Laplacian regularization term to capture the local relationship between drug pairs, and the fourth term is the Laplacian regularization term to capture the local relationship between target pairs.

As can be seen from Equation (12), DLGrLMF models the interaction probability between a drug-target pair by a logistic function and decomposes the probability matrix into drug-specific and target-specific latent vectors. In DLGrLMF, a biologically validated drug-target pair is treated as positive examples, while an unknown pair is treated as a negative example. In such a manner, DLGrLMF assigns higher weights to positive observations than negatives. Since the positive pairs are biologically validated and thus usually more trustworthy while the negative pairs could contain potential DTIs and are thus unreliable, our method can fully exploit the useful information in validated interaction pairs.

In this work, we use gradient descent to optimize Equation (12). Supposing the objective function is denoted as *ℱ*, then the partial derivatives of *ℱ* with respect to **U** and **V** can be obtained as follows:

(13) $$\begin{array}{c} \frac{\partial F}{\partial U}=PV+\left(n-1\right)\left(A⨀P\right)V-nAV+\alpha \left(I_{m}+L_{D}\right)U,\\ \frac{\partial F}{\partial V}=P^{T}U+\left(n-1\right)\left(A^{T}⨀P^{T}\right)U-nA^{T}U+\beta \left(I_{n}+L_{T}\right)V, \end{array}$$

where ⨀ denotes the Hadamard product of two matrices. Each element of **P** (i.e., *p*_*i*_*j*) is formulated by Equation (1), which denotes the probability of interaction between drug *i* and target *i*. **U** and **V** are randomly initialized. During the optimization process, **U** and **V** are updated until to be stable. After we get the final solution $\hat{U}$ and $\hat{V}$, the final probability of interaction between drug *i* and target *j* can be calculated as follows:

(14) $$\begin{array}{c} {\hat{p}}_{ij}=\frac{e^{\left({\hat{u}}_{i}{{\hat{v}}_{j}}^{T}\right)}}{\left(1+e^{\left({\hat{u}}_{i}{{\hat{v}}_{j}}^{T}\right)}\right)}. \end{array}$$

## 3. Experimental Results

### 3.1. Evaluation Metrics

In order to validate the efficacy of our proposed method, experiments on five datasets mentioned in Section 2.1 are conducted. Similar to several previous works [49–51], two evaluation metrics including precision-recall (PR) curves and the Area Under the Precision-Recall curves (AUPR) [52] are utilized for performance evaluation. Since we intend to avoid incorrect predictions being recommended by the prediction algorithms [52], AUPR is desirable for evaluation because it can penalize the false positives more.

### 3.2. Experiment Settings

In our experiments, we use other six drug-target interaction prediction techniques for performance comparison, they are bipartite local model using neighbor-based interaction-profile inferring (BLMNII) [49], weighted nearest neighbor profile (WNN) [50], collaborative matrix factorization (CMF) [31], graph regularized matrix factorization (GRMF) [51], neighborhood regularized logistic matrix factorization (NRLMF) [45] and label propagation with linear neighborhood information (LPLNI) [53], and dual Laplacian graph regularized matrix completion for drug-target interaction prediction (DLGRMC). For each method, we perform 5 repetitions of 10-fold cross-validation (CV) on different datasets. In each repetition, the observed DTI indicator matrix **A** was divided into 10 folds. Then, each fold was selected for testing while the remaining 9 folds were used for training; the final AUPR score was the average results over 5 repetitions.

Similar to previous works [31, 54, 55], we conduct CV under the following three different settings:

- CV_1_: CV on drug-target pairs—we randomly select some entries from **A** (i.e., drug-target pairs) for testing, which refers to test the efficacy of the DTI prediction method for new (unknown) drug-target pairs
- CV_2_: CV on drugs—we randomly select several rows in **A** (i.e., drugs) for testing, which refers to the DTI prediction for new drugs
- CV_3_: CV on targets—we randomly select a portion of columns in **A** (i.e., targets) for testing; this setting refers to the DTI prediction for new targets

As to CV_1_, CV_2_, and CV_3_, we use 90% of entries in **A**, 90% of rows in **A**, and 90% of columns in **A** as training data and the remaining data as testing data in each round, respectively.

### 3.3. DTI Prediction Results

**Figure 1:**
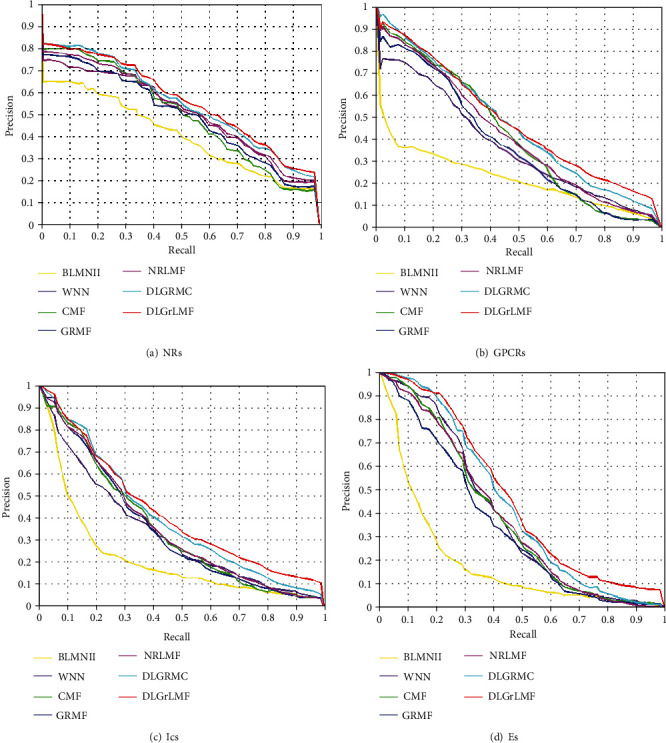
The PR curves of different methods on different datasets.

In Tables 2–4, we show the predicted AUPR values of different methods on different datasets under varying CV settings. As can be seen, our proposed DLGrLMF consistently outperforms other methods on all of the datasets. Considering that the drug discovery and development aim to serve the treatment of disease, in order to predict new targets which the drugs react, we plot the precision-recall (PR) curves of the results under CV_3_ for all of the datasets. The PR curves are shown in Figure 1; the results also demonstrate the superiority of our proposed DLGrLMF.

### 3.4. Case Study

In order to validate the capacity of DLGrLMF for potential DTI prediction, we randomly choose a drug from each dataset and report the top 10 predicted interactions of different methods under CV_3_. The predicted results are reported in Tables 5–8. As can be seen from the results, our proposed DLGrLMF can successfully predict a larger amount of the experimental validated DTIs when compared with other methods, which also indicates that DLGrLMF is capable of predicting novel DTIs for drug development.

## 4. Discussion and Conclusions

In this paper, we propose a novel dual Laplacian graph regularized logistic matrix factorization model for drug-target interaction prediction, i.e., DLGrLMF. Specifically, DLGrLMF regards the task of drug-target interaction prediction as a weighted logistic matrix factorization problem, in which the experimentally validated interactions are allocated with larger weights. Meanwhile, by considering that drugs with similar chemical structure should have interactions with similar targets and targets with similar genomic sequence similarity should in turn have interactions with similar drugs, the drug pairwise chemical structure similarities as well as the target pairwise genomic sequence similarities are fully exploited to serve the matrix factorization problem by using a dual Laplacian graph regularization term. By performing extensive experiments, the efficacy of the proposed method can be well validated, and case studies demonstrate that the proposed method is powerful to predict potential novel drug-target interactions.

In addition, experimental results also demonstrate that there is still much room for improvement since there also exists missed interactions in case studies. In this work, only one type of representation for drugs or targets is used. In practical, each drug/target is often with multiple representations. For example, a drug can be represented by its chemical structure or by its chemical response in different cells. A protein target can be represented by its sequence or by its gene expression values in different cells. In our future work, we will try to integrate multiple representations for drug-target interaction prediction and we believe that the prediction results can be improved with a large margin.

## Figures and Tables

**Table 1:** The statistics of drugs, targets, and interactions in each dataset.

Datasets	NRs	GPCRs	ICs	Es
No. of drugs	54	223	210	445
No. of targets	26	95	204	664
No. of interactions	90	635	1476	2926
Average no. of drugs/target	3.46	6.68	7.24	4.41
Average no. of targets/drug	1.67	2.85	7.03	6.58
Sparsity of the interaction matrix (%)	93.59	97	96.55	99.01

**Table 2:** Average AUPR values of different methods on the four datasets under CV_1_ setting (the values following the symbol “±” are the standard deviations of 5 repetition results).

Methods	NRs	GPCRs	Ics	Es
BLMNII	0.642 ± 0.039	0.482 ± 0.017	0.647 ± 0.012	0.622 ± 0.014
WNN	0.568 ± 0.023	0.589 ± 0.021	0.586 ± 0.016	0.593 ± 0.015
CMF	0.578 ± 0.037	0.676 ± 0.012	0.855 ± 0.006	0.804 ± 0.004
GRMF	0.595 ± 0.024	0.677 ± 0.013	0.368 ± 0.017	0.325 ± 0.013
NRLMF	0.677 ± 0.035	0.679 ± 0.014	0.887 ± 0.012	0.846 ± 0.005
DLGRMC	0.697 ± 0.025	0.705 ± 0.016	0.897 ± 0.016	0.877 ± 0.006
DLGrLMF	0.713 ± 0.020	0.724 ± 0.012	0.908 ± 0.015	0.896 ± 0.006

**Table 3:** Average AUPR values of different methods on the four datasets under CV_2_ setting (the values following the symbol “±” are the standard deviations of 5 repetition results).

Methods	NRs	GPCRs	Ics	Es
BLMNII	0.429 ± 0.046	0.309 ± 0.022	0.288 ± 0.027	0.287 ± 0.027
WNN	0.505 ± 0.503	0.287 ± 0.017	0.238 ± 0.035	0.236 ± 0.035
CMF	0.467 ± 0.056	0.357 ± 0.015	0.266 ± 0.033	0.269 ± 0.030
GRMF	0.483 ± 0.052	0.355 ± 0.019	0.287 ± 0.025	0.286 ± 0.024
NRLMF	0.543 ± 0.052	0.364 ± 0.017	0.347 ± 0.033	0.346 ± 0.034
DLGRMC	0.576 ± 0.053	0.375 ± 0.015	0.367 ± 0.023	0.367 ± 0.023
DLGrLMF	0.587 ± 0.018	0.395 ± 0.011	0.379 ± 0.014	0.382 ± 0.019

**Table 4:** Average AUPR values of different methods on the four datasets under CV_3_ setting (the values following the symbol “±” are the standard deviations of 5 repetition results).

Methods	NRs	GPCRs	Ics	Es
BLMNII	0.414 ± 0.041	0.335 ± 0.013	0.203 ± 0.015	0.164 ± 0.014
WNN	0.515 ± 0.023	0.365 ± 0.006	0.320 ± 0.013	0.386 ± 0.015
CMF	0.486 ± 0.037	0.404 ± 0.008	0.357 ± 0.012	0.378 ± 0.005
GRMF	0.518 ± 0.028	0.364 ± 0.013	0.345 ± 0.019	0.348 ± 0.012
NRLMF	0.494 ± 0.046	0.412 ± 0.045	0.356 ± 0.014	0.397 ± 0.015
DLGRMC	0.528 ± 0.023	0.415 ± 0.016	0.364 ± 0.018	0.414 ± 0.013
DLGrLMF	0.538 ± 0.022	0.432 ± 0.013	0.388 ± 0.014	0.425 ± 0.011

**Table 5:** The top 10 interacted targets of drug “D00094” in dataset NRs predicted by different methods (“√” denotes experimentally validated targets, and “×” denotes nonvalidated targets).

Rank	Targets predicted by different methods						
	BLMNII	WNN	CMF	GRMF	NRLMF	DLGRMC	DLGrLMF
1	hsa5914 (√)	hsa190 (√)	hsa6096 (√)	hsa6257 (√)	hsa5915 (√)	hsa5914 (√)	hsa5914 (√)
2	hsa5915 (√)	hsa6257 (√)	hsa6257 (√)	hsa5915 (√)	hsa190 (√)	hsa5915 (√)	hsa5915 (√)
3	hsa6257 (√)	hsa5915 (√)	hsa5915 (√)	hsa6256 (√)	hsa6096 (√)	hsa190 (√)	hsa6096 (√)
4	hsa190 (√)	hsa6256 (√)	hsa190 (√)	hsa190 (√)	hsa5914 (√)	hsa6096 (√)	hsa190 (√)
5	hsa6258 (√)	hsa190 (√)	hsa6256 (√)	hsa6258 (√)	hsa6097 (√)	hsa6257 (√)	hsa5256 (√)
6	hsa6097 (√)	hsa6097 (√)	hsa5916 (√)	hsa5916 (√)	hsa6258 (√)	hsa6256 (√)	hsa6257 (√)
7	hsa2099 (×)	hsa5916 (√)	hsa2104 (×)	hsa5915 (√)	hsa5916 (√)	hsa6258 (√)	hsa6258 (√)
8	hsa4306 (×)	hsa2908 (×)	hsa2421 (×)	hsa2101 (×)	hsa6257 (√)	hsa5916 (√)	hsa5916 (√)
9	hsa5465 (×)	hsa2104 (×)	hsa4306 (×)	hsa2104 (×)	hsa367 (×)	hsa2099 (×)	hsa6097 (√)
10	hsa2104 (×)	hsa2421 (×)	hsa9970 (×)	hsa5465 (×)	hsa4306 (×)	hsa2908 (×)	hsa9970 (×)

**Table 6:** The top 10 interacted targets of drug “D00255” in dataset GPCRs predicted by different methods (“√ “denotes experimentally validated targets, and “×” denotes nonvalidated targets).

Rank	Targets predicted by different methods						
	BLMNII	WNN	CMF	GRMF	NRLMF	DLGRMC	DLGrLMF
1	hsa147 (√)	hsa150 (√)	hsa151 (√)	hsa155 (√)	hsa155 (√)	hsa147 (√)	hsa155 (√)
2	hsa148 (√)	hsa146 (√)	hsa146 (√)	hsa150 (√)	hsa147 (√)	hsa155 (√)	hsa151 (√)
3	hsa146 (√)	hsa155 (√)	hsa147 (√)	hsa151 (√)	hsa146 (√)	hsa151 (√)	hsa150 (√)
4	hsa150 (√)	hsa153 (√)	hsa148 (√)	hsa147 (√)	hsa150 (√)	hsa150 (√)	hsa147 (√)
5	hsa1812 (×)	hsa154 (√)	hsa155 (√)	hsa154 (√)	hsa148 (√)	hsa146 (√)	hsa148 (√)
6	hsa2550 (×)	hsa1234 (×)	hsa154 (√)	hsa1268 (×)	hsa2550 (×)	hsa154 (√)	hsa154 (√)
7	hsa2913 (×)	hsa1241 (×)	hsa2911 (×)	hsa135 (×)	hsa3361 (×)	hsa1128 (×)	hsa153 (√)
8	hsa5739 (×)	hsa3354 (×)	hsa1241 (×)	hsa2911 (×)	hsa5729 (×)	hsa2911 (×)	hsa2911 (√)
9	hsa7201 (×)	hsa7201 (×)	hsa3354 (×)	hsa57105 (×)	hsa9052 (×)	hsa3269 (×)	hsa3354 (×)
10	hsa552 (×)	hsa6751 (×)	hsa6751 (×)	hsa886 (×)	hsa2911 (×)	hsa3352 (×)	hsa1268 (×)

**Table 7:** The top 10 interacted targets of drug “D00110” in dataset ICs predicted by different methods (“√” denotes experimentally validated targets, and “× “denotes nonvalidated targets).

Rank	Targets predicted by different methods						
	BLMNII	WNN	CMF	GRMF	NRLMF	DLGRMC	DLGrLMF
1	hsa6336 (√)	hsa11280 (√)	hsa6530 (√)	hsa6532 (√)	hsa6529 (√)	hsa6331 (√)	hsa6530 (√)
2	hsa6532 (√)	hsa6530 (√)	hsa6532 (√)	hsa11280 (√)	hsa6532 (√)	hsa6336 (√)	hsa6532 (√)
3	hsa6530 (√)	hsa6529 (√)	hsa11280 (√)	hsa6336 (√)	hsa6336 (√)	hsa6530 (√)	hsa6336 (√)
4	hsa11280 (√)	hsa6331 (√)	hsa6529 (√)	hsa6336 (√)	hsa6331 (√)	hsa6532 (√)	hsa6331 (√)
5	hsa6529 (√)	hsa6532 (√)	hsa6331 (√)	hsa6530 (√)	hsa11280 (√)	hsa11280 (√)	hsa11280 (√)
6	hsa2554 (×)	hsa2554 (×)	hsa6336 (√)	hsa6529 (√)	hsa9312 (×)	hsa6529 (√)	hsa6529 (√)
7	hsa2901 (×)	hsa9177 (×)	hsa2901 (×)	hsa1137 (×)	hsa93589 (×)	hsa1141 (×)	hsa11254 (√)
8	hsa3748 (×)	hsa773 (×)	hsa27012 (×)	hsa9312 (×)	hsa23704 (×)	hsa1137 (×)	hsahsa10060 (×)
9	hsa1134 (×)	hsa8514 (×)	hsa8973 (×)	hsa3762 (×)	hsa2892 (×)	hsa9312 (×)	hsa9132 (×)
10	hsa9177 (×)	hsa9311 (×)	hsa2560 (×)	hsa1139 (×)	hsa3756 (×)	hsa93589 (×)	hsa9311 (×)

**Table 8:** The top 10 interacted targets of drug “D00002” in dataset Es predicted by different methods (“√” denotes experimentally validated targets, and “×” denotes nonvalidated targets).

Rank	Targets predicted by different methods						
	BLMNII	WNN	CMF	GRMF	NRLMF	DLGRMC	DLGrLMF
1	hsa216 (√)	hsa108 (√)	hsa1725 (×)	hsa196883 (√)	hsa191 (√)	hsa191 (√)	hsa191 (√)
2	hsa108 (√)	hsa1725 (×)	hsa108 (√)	hsa191 (√)	hsa196883 (√)	hsa1725 (×)	hsa107 (√)
3	hsa1725 (×)	hsa191 (√)	hsa2936 (√)	hsa7498 (√)	hsa108 (√)	hsa196883 (√)	hsa108 (√)
4	hsa2746 (√)	hsa3939 (√)	hsa2639 (√)	hsa3033 (√)	hsa3292 (√)	hsa108 (√)	hsa6652 (√)
5	hsa196883 (√)	hsa3292 (√)	hsa115 (√)	hsa108 (√)	hsa3615 (√)	hsa2936 (√)	hsa64802 (√)
6	hsa7015 (×)	hsa349565 (√)	hsa2597 (√)	hsa7299 (×)	hsa3939 (√)	hsa3033 (√)	hsa4190 (√)
7	hsa4594 (×)	hsa34 (×)	hsa3156 (×)	hsa84152 (×)	hsa3818 (×)	hsa349565 (√)	hsa196883 (√)
8	hsa3035 (×)	hsa8435 (×)	hsa51095 (×)	hsa590 (×)	hsa5536 (×)	hsa339221 (×)	hsa3376 (×)
9	hsa306 (×)	hsa51095 (×)	hsa90 (×)	hsa3156 (×)	hsa34 (×)	hsa3156 (×)	hsa34 (×)
10	hsa8435 (×)	hsa306 (×)	hsa761 (×)	hsa34 (×)	hsa90 (×)	hsa3991 (×)	hsa3156 (×)

## Data Availability

The datasets used in this work are publicly available at http://web.kuicr.kyoto-u.ac.jp/supp/yoshi/drugtarget/.
